# The LCP Family Protein, Psr, Is Required for Cell Wall Integrity and Virulence in *Streptococcus agalactiae*

**DOI:** 10.3390/microorganisms10020217

**Published:** 2022-01-20

**Authors:** Atefeh Rajaei, Hannah M. Rowe, Melody N. Neely

**Affiliations:** 1 Molecular and Biomedical Sciences Department, University of Maine, Orono, ME 04469, USA; atefeh.rajaei@maine.edu; 2 Department of Microbiology, Oregon State University, Corvallis, OR 97331, USA; hannah.rowe@oregonstate.edu

**Keywords:** *Streptococcus agalactiae*, LCP family proteins, cell wall integrity, pathogenesis

## Abstract

A robust cell envelope is the first line of protection for an infecting pathogen when encountering the immune defense of its host. In Gram-positive organisms, LytR-CpsA-Psr (LCP) family proteins play a major role in the synthesis and assembly of the cell envelope. While these proteins could be considered for potential new drug targets, not enough is known about how they function to support the integrity of the cell wall. *Streptococcus agalactiae* (group B streptococcus or GBS) is known to encode at least three LCP family proteins, including CpsA, LytR (BrpA) and Psr. Using strains of GBS that have mutations in two of the three LCP proteins, we were able to determine a role for these proteins in GBS cell wall integrity. The results presented here demonstrate that the absence of Psr results in a decreased growth rate, decreased viability over time, inconsistent cocci morphology and diminished cell wall integrity, as well as an increased penicillin susceptibility, decreased capsule levels and attenuation in virulence in a zebrafish model of infectious disease. A strain that is missing two of the LCP family proteins, CpsA and Psr, exhibits an increase in these defective phenotypes, indicating that CpsA and Psr are partially redundant in function.

## 1. Introduction

*Streptococcus agalactiae* (GBS) is a commensal organism that is typically present in the lower gastrointestinal and reproductive tract of 15–30% of individuals [1,2,3]. GBS can cause invasive infection in the most vulnerable groups of the population, including the elderly with underlying health conditions, adults with compromised immune systems and, most devastatingly, in neonates, due to their underdeveloped immune system at birth. For many decades, GBS has been identified as the leading cause of bacterial infection leading to sepsis, meningitis and death among newborns [4]. GBS infections in newborns are classified into two different forms based on the time of onset, with early-onset disease occurring within the first 7 days of life. Late-onset disease occurs within 7–90 days after birth and is most commonly transferred from the colonized mother to the baby during pregnancy or labor [5,6]. The current preventative measure for neonatal GBS disease is screening pregnant women for GBS colonization during weeks 35–37 of pregnancy and the intravenous administration of antibiotics to the colonized mother during labor [6]. Though this practice has dramatically reduced the occurrence of early-onset GBS disease, it does not prevent late-onset GBS disease, as it is not always transmitted from the colonized mother during labor and is rather acquired in the hospital or through community transmission [5,7]. Additionally, studies have shown that neonatal exposure to antibiotics dramatically affects the gut microbiome and may lead to lifelong immune system deficiencies and conditions such as obesity, inflammatory bowel disease, allergies, juvenile rheumatoid arthritis, wheezing and asthma [8,9,10,11,12]. Moreover, multiple GBS clinical isolates have become resistant to specific antibiotics [13,14,15,16], causing concerns about the future of GBS infection treatment. Therefore, there is an urgent need for the development of new therapeutic strategies to help combat GBS infections.

The development of new antimicrobials requires identification and a mechanistic understanding of new bacterial targets. The LytR-CpsA-Psr (LCP) family of proteins are potential antimicrobial targets for Gram-positive pathogens, including GBS. LCP proteins have been examined in pathogens such as *Staphylococcus aureus*, *Streptococcus pneumoniae* and *Streptococcus mutans*; and are involved in the biosynthesis and maintenance of the cell wall and capsule [17,18,19,20]. LCP family protein members all contain at least one transmembrane region, with the majority of the protein residing on the extracellular portion of the plasma membrane. In GBS, three LCP proteins—CpsA, LytR (BrpA) and Psr—have been identified. CpsA is a multifunctional protein, with the N-terminus able to act as a DNA binding protein that binds with specificity to the promoter of the capsular polysaccharide (cps) operon [21,22] and is also proposed to be the ligase that attaches newly assembled polysaccharide capsule onto the cell wall [18,23]. LytR (BrpA) regulates biofilm formation and influences GBS virulence [24]. The exact role of Psr, however, has not yet been characterized in GBS.

In this study, we demonstrate the role of Psr in the maintenance of the cellular morphology, cell wall integrity and virulence of GBS. A loss of Psr results in a growth deficiency, increased autolysis in the presence of compounds that disrupt structural integrity, decreased capsular polysaccharide on the cell surface and the attenuation of virulence in a zebrafish model of infectious disease.

## 2. Materials and Methods

### 2.1. Bacterial Strains and Growth Conditions

The streptococcal strains *Streptococcus agalactiae* GBS 515 and the CpsA deletion strain (*cpsA*) were described previously [22,25]. Streptococcal liquid cultures were grown statically in closed tubes in THY medium (Todd Hewitt Broth, Dot Scientific; Burton, MI, USA) +0.2% yeast extract at 37 °C. The Psr mutant and the CpsA-Psr double mutant strains were supplemented with erythromycin (2 μg/mL). The Psr mutant strain was constructed by amplifying a 490 bp region of GBS 515 genomic DNA using primers (5′ GBS Psr ins *EcoR*I CCGGAATTCGCTAAATCATCATGAAGAGC- and 3′ GBS Psr ins *Pst*I- AAAACTGCAGTTAAGCTCCCATCAACAGC) and ligated into the *Eco*RI and *Pst*I sites of plasmid pUC19-Erm [26]. The plasmid was electrotransformed as described previously into GBS 515 and its isogenic *cpsA* deletion strain [25]. Single crossover recombinants were selected by growth on erythromycin (2 μg/mL) on THY (Dot Scientific; Burton, MI, USA) agar plates supplemented with 0.2% yeast extract and 1.4% bacteriological agar (Dot Scientific; Burton, MI, USA).

### 2.2. Growth Curve

Overnight cultures were subcultured 1:100 into 20 mL of fresh THY medium and grown statically at 37 °C. Growth of cultures was monitored by measuring and recording the OD_600_ once every hour for 10 h using a spectrophotometer. Values represented are averages of 3 biological replicates.

### 2.3. Antibiotic Assay

Overnight cultures were subcultured 1:100 in 20 mL of fresh Todd Hewitt Broth (Dot Scientific; Burton, MI, USA) +0.2% yeast extract (VWR Scientific; Radnor, PA, USA) and normalized to 0.01 OD_600_. Cells (150 µL) were added to clear, flat-bottom 96-well plates in triplicate and supplemented with 0 µg/mL (untreated) and 0.03 µg/mL of penicillin. Plates were sealed and incubated at 37 °C for 24 h. Contents of each well were mixed by pipetting, then OD_600_ measurements were taken via plate reader (Bio Tek, Synergy 2, Agilent Technologies; Santa Clara, CA, USA). Viable counts were determined by plating serial 10-fold dilutions of samples 24 h post treatment on THY-agar plates. Values represented are averages of 4 biological replicates.

### 2.4. Live/Dead Assay

The live/dead assay was performed according to LIVE/DEAD™ *Bac*Light™ Bacterial Viability Kit (Invitrogen, Thermo Fisher Scientific, Waltham, MA, USA) protocol. Briefly, cells at mid-log growth phase (0.3 OD_600_) and at stationary growth phase (overnight cultures) were collected and washed once and resuspended in 0.85% NaCl. Five microliters of cells and 5 µL of 2× stain were thoroughly mixed by pipetting and incubated at room temperature for 15 min while protected from light. Stained cells (7 µL) were pipetted onto a glass slide with a coverslip and observed via fluorescent microscopy using Zeiss Axioscope 40 (Carl Zeiss Microscopy, White Plains, NY, USA) and a FITC filter at 1000× magnification and imaged with Axiovision 4.7 imaging software. Images were quantified by recording chain lengths for each strain using 10 fields of view per sample. 

### 2.5. Triton X-100 Susceptibility

Overnight cultures were subcultured and grown statically at 37 °C. Cells were collected at mid-log growth phase and normalized to 0.3 OD_600_ in PBS. Samples were treated with 0.01% final concentration of Triton X-100 (Thermo Fisher Scientific, Waltham, MA, USA) in PBS for 2 h at 37 °C. Serial 10-fold dilutions were plated on THY-agar plates, incubated at 37 °C overnight and compared to untreated cells. Values represented are averages of 6 biological replicates.

### 2.6. Lysozyme Susceptibility

Overnight cultures were subcultured and grown statically at 37 °C. Cells were collected at mid-log growth phase and normalized to 0.3 OD_600_ in PBS. Samples were treated with 1 mg/mL final concentration of lysozyme (Sigma-Aldrich, St. Louis, MO, USA) in PBS for 24 h. Serial 10-fold dilutions were plated on THY-agar plates, incubated overnight and compared to untreated cells. Values represented are averages of 4 biological replicates.

### 2.7. Capsule Measurement by Buoyant Density

Buoyant density was determined using linear Percoll (Sigma-Aldrich, St. Louis, MO, USA) gradients as described previously [27]. Briefly, 4 mL Percoll, supplemented with 0.15 M NaCl and diluted to low (1.085 g/cm^3^) density, was carefully overlaid onto 4 mL Percoll diluted to high (1.120 g/cm^3^) density in a 10 mL tube and placed at a 15° angle overnight to allow for formation of a continuous linear gradient. Next day, gradients were gently placed vertically and allowed to settle at least 30 min prior to centrifugation. Overnight bacterial cultures were normalized to an 0.6 OD_600_ in 1 mL and concentrated to a volume of 100 μL in phosphate-buffered saline. Culture was carefully added to top of gradient and gradients were centrifuged 30 min at room temperature at 5000 rpm in a swinging bucket. Distance traveled in the gradient was measured from the bottom of the meniscus to the center of the cell band, and density was determined by calculation of a linear curve from distance traveled by beads of known density (Sigma-Aldrich, St. Louis, MO, USA). Values represented are averages of 3 biological replicates.

### 2.8. Measurement of Capsule in Supernatants

Overnight cultures were subcultured and grown statically at 37 °C. Cells were collected at mid-log growth phase and normalized to 0.3 OD_600_ in TBS. Culture supernatants were serially diluted and 5 µL was pipetted onto 0.45 μm PVDF membrane and allowed to air dry. The membrane was then treated with 5% blocking buffer at 4 °C for 1 h on an orbital shaker. Membrane was then treated with 5% blocking buffer + 1:10,000 final concentration of primary antibody (Rabbit α-GBS serotype 1a) at 4 °C overnight on an orbital shaker. The next morning, the membrane was washed 3× with TBST for 5 min each, then treated with 5% blocking buffer with a 1:5000 final concentration of goat anti-rabbit IgG-AP (Thermo Fisher Scientific, Waltham, MA, USA) for 1 h, then washed again 3× with TBST for 5 min each. The membrane was then treated with 4 mL BCIP/NBT substrate (Sigma-Aldrich, St. Louis, MO, USA) until spots became visible. Experiment was repeated 3 times.

### 2.9. Chain Length Analysis

Overnight cultures were pipetted onto a microscope slide with a coverslip and observed using a Zeiss Axioscope 40 bright field microscopy at 1000× magnification and imaged with Axiovision 4.7. Chain lengths were quantified using 10 fields of view per sample. A minimum of 2500 cocci per strain were quantified.

### 2.10. Fluorescent Vancomycin Assay

Overnight cultures were subcultured and grown statically at 37 °C. Cells were collected at mid-log growth phase and normalized to 0.3 OD_600_ in PBS and concentrated to 1.0 OD_600_. Ten microliters of samples were treated with 1 μg/μL BODIPY_FL vancomycin (Thermo Fisher Scientific, Waltham, MA, USA) and incubated for 10 min at 37 °C while protected from light. Treated samples were then washed 3× with PBS and resuspended in 3 μL PBS. Samples were observed and imaged using confocal microscopy. Micrographs are representative of at least 15 images per strains, recorded from 2 biological replicates.

### 2.11. Zebrafish Infection

Cells were collected at mid-log growth phase, normalized to 0.225 OD_600_ (~1 × 10^8^ cfu/mL) and washed 2× with PBS. Zebrafish (Zf5) were anesthetized with 0.32 mg/mL tricaine and injected at 72 h post fertilization with 1 nano-liter (~100 cfu) of bacteria in the yolk sack. Injections were performed via PV830 pneumatic picopump microinjector (World Precision Instruments; Sarasota, FL, USA) and Stemi 508 microscope. Fish were then observed for 72 h for survival post infection. Dosages were verified by plating serial 10-fold dilutions of 100 cfu samples. Kaplan–Meier curves were generated using GraphPad Prism 9. Values represented are averages of 4 biological replicates with a minimum of 105 total zebrafish injected per strain. All experiments were carried out according to the University of Maine IACUC committee approved protocol A-2020-02-01.

### 2.12. Statistical Analysis

Statistical analysis for Triton-X-100-induced autolysis, lysozyme-induced autolysis, penicillin susceptibility and buoyant density assays was conducted using Microsoft Excel data analysis tool, by first conducting an F-test to determine variance followed by a *t*-test to determine significance. *p* < 0.05. Statistical analysis for zebrafish infection was conducted using Prism 9 version 9.1.2 log-rank (Mantel–Cox) test. 

## 3. Results

### 3.1. Loss of Psr Results in a Growth Deficiency

**Figure 1:**
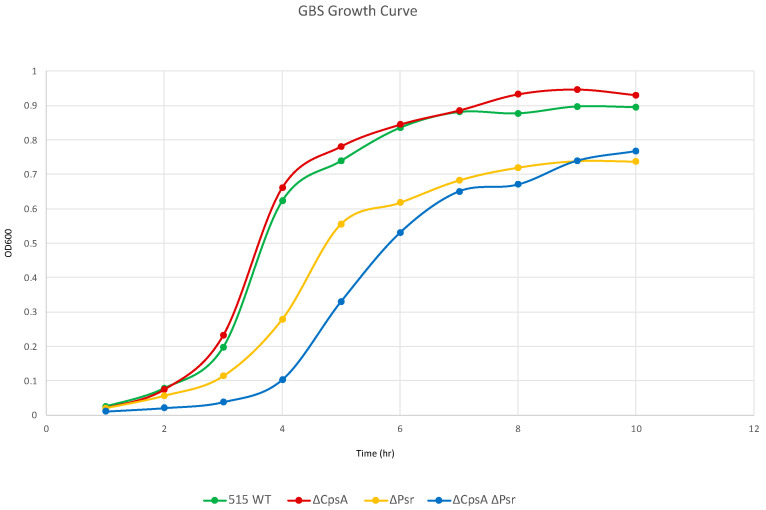
GBS growth decreases in the absence of Psr. Overnight cultures were subcultured 1:100 in fresh media and grown statically at 37 °C. OD_600_ was measured via spectrophotometer at the specified time points. Graph is representative of three separate growth curves.

As LCP proteins are suggested to play a role in cell envelope synthesis and maintenance [17,18,23], we wanted to determine if strains lacking Psr exhibited a change in overall cell growth. The growth rate of the wild type GBS, ΔCpsA, ΔPsr and ΔCpsA-ΔPsr strains in THY liquid culture was determined. Whereas wild type GBS and ΔCpsA GBS strains grow similarly, the ΔPsr and ΔCpsA-ΔPsr mutant strains grow at a considerably slower rate (Figure 1). When comparing the ΔCpsA and the ΔCpsA-ΔPsr mutant strains, whereas the growth of the ΔCpsA strain is not affected, the double mutant strain shows the slowest growth rate of all four strains. These results suggest that, in the absence of Psr, CpsA may be partially substituting for its functions; therefore, in the absence of both genes, the growth rate substantially decreases.

### 3.2. CpsA-Psr Double Mutant Strain Is Significantly Less Viable over Time

**Figure 2:**
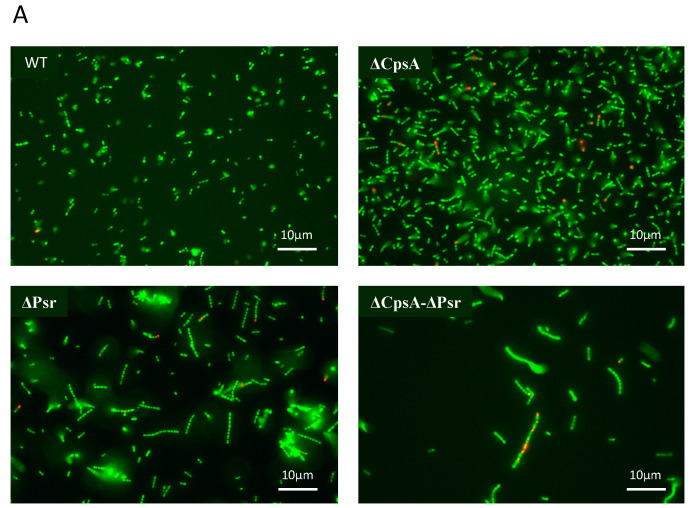

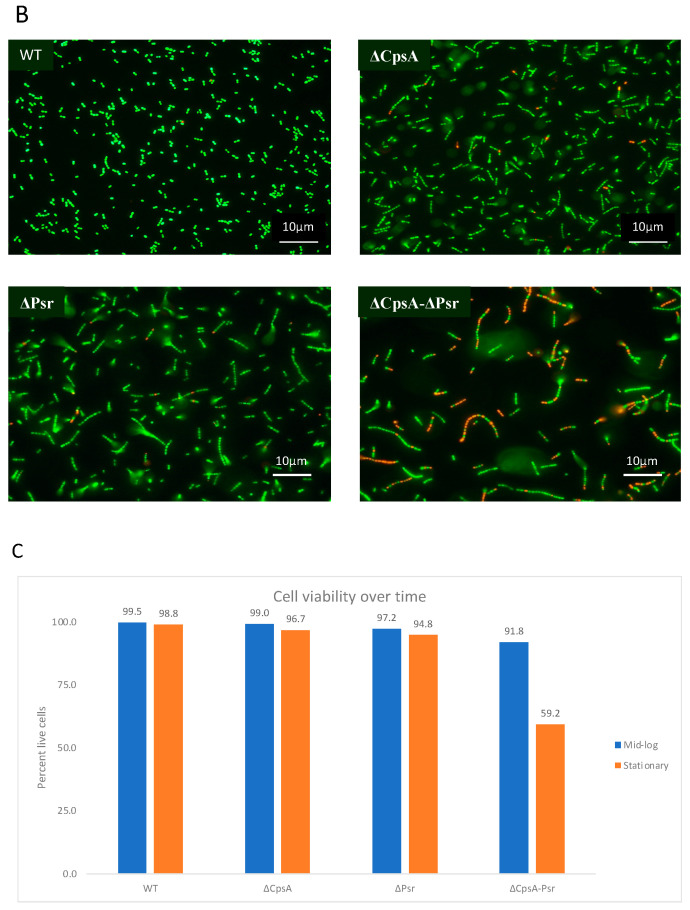
GBS cell viability is significantly affected at stationary phase. GBS strains were grown to mid-log and stationary phase in THY medium, normalized to OD600: 0.6 in 0.85% NaCl and treated with live/dead stain. (**A**) Images of cells grown to mid-log phase, where green cells represent live cells and red cells represent dead cells. (**B**) Images of cells grown to stationary phase, where green cells represent live cells and red cells represent dead cells. (**C**) Percentage of live cells were quantified. Micrographs of each strain were imaged and 10 fields of view per strain were used to quantify percentage of live cells.

To determine if the decreased growth rate observed with the ΔPsr and ΔCpsA-ΔPsr mutants is a result of missing components needed for cell growth or a loss of viability of cells overtime, live/dead assays were performed on all strains at the mid-log and stationary phase of growth. The live/dead assay determines cell viability as a function of the membrane integrity of the cell. Therefore, live cells stained in this assay fluoresce green from the SYTO9 dye, whereas cells that are thought to be dead or dying will stain red from propidium iodide. Although no significant cell death was observed at the mid-log growth phase (Figure 2A,C) for any of the four strains, a surprising 41% cell death was observed for the ΔCpsA-ΔPsr double mutant strain at the stationary phase (Figure 2B,C).

### 3.3. Loss of CpsA and Psr Results in an Increase in Bacterial Chain Length

**Figure 3:**
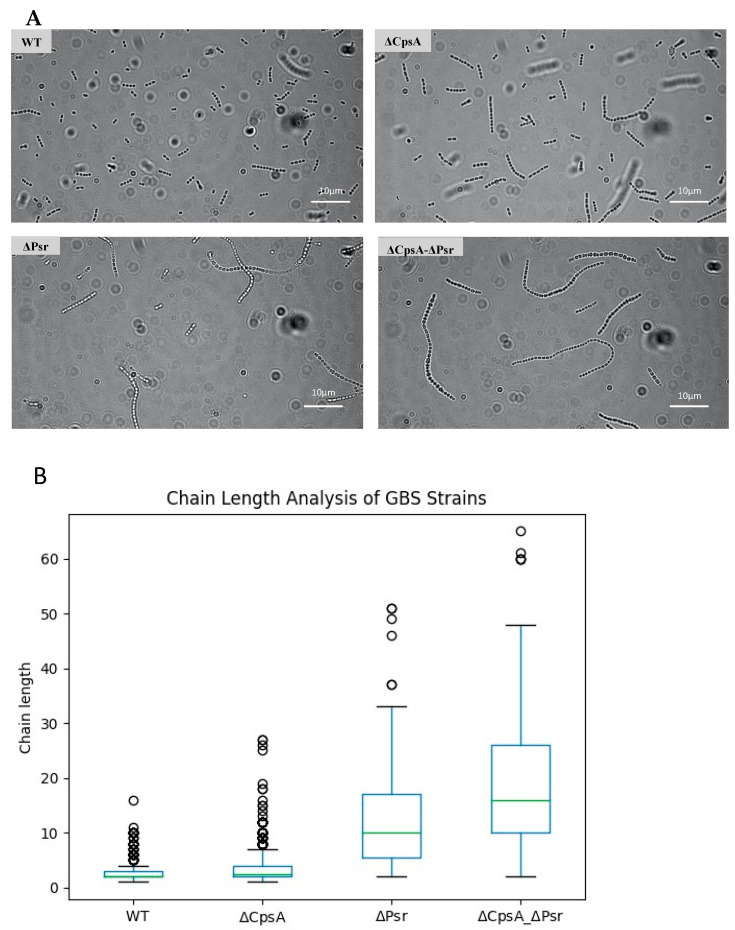
GBS chain length increases in the absence of CpsA and Psr. Overnight cultures were mounted on microscope slides and visualized via brightfield microscopy. (**A**) Micrograph images of each of the four GBS strains pipetted onto a glass slide. (**B**) A quantitative representation of the chain length analysis. Images using 10 fields of view from each strain were used to quantify the number of cocci in each chain, with green line indicative of median chain length.

As the Psr protein is thought to be directly involved in the production of the peptidoglycan cell wall and an increase in the chain length has been associated with cell wall abnormalities [17], we wanted to determine if the absence of Psr would result in a change in the cocci chain length. Using a bright field microscopy of cultures, we visualized and quantified the chain lengths of each strain. The chain length analysis revealed that the wild type strain normally grow as chains of 2–6 cocci, whereas the majority of the chains in the ΔCpsA strain normally have 4–10 cocci (Figure 3A,B). The ΔPsr mutant strain exhibits longer chain lengths of approximately 6–30 cocci, whereas the double mutant demonstrates chain lengths of as long as 70 cocci (Figure 3A,B).

### 3.4. Loss of Psr Results in Changes in Cell Wall Integrity 

**Figure 4:**
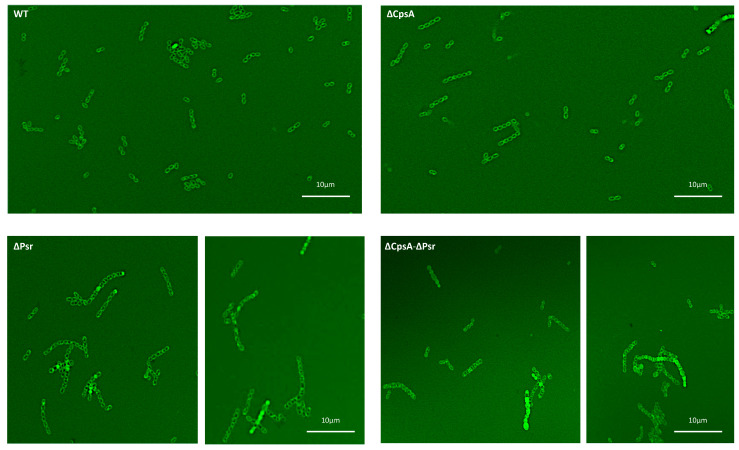
Bacterial cocci morphology changes in the absence of Psr. Overnight cultures were incubated with fluorescent vancomycin and imaged via confocal microscopy. WT and the ΔCpsA strains exhibit shorter chains, uniform cocci morphology and intact cell walls, whereas the ΔPsr and ΔCpsA-ΔPsr mutant strains exhibit longer chain lengths, leaky cell walls and anomalous cell morphologies. Micrographs are representative of images recorded from two assays repeated on 2 different days.

To determine if the absence of the Psr protein from the cell causes a change in the cell wall morphology, we performed fluorescent vancomycin assays. Fluorescent vancomycin binds to newly formed cell walls and is a green fluorescent analog of vancomycin, allowing for the visualization of cell morphology using fluorescent microscopy. An observation of the cells tagged with fluorescent vancomycin revealed a possible change in the cell wall integrity in all mutants, but most notably in the ΔPsr and ΔCpsA-ΔPsr mutant strains. Since the tagged fluorescent dye binds to the cell wall, an intact cell wall would only stain around the outside of the cell. If the stain is observed inside the cell, then this suggests a loss of cell wall integrity, allowing for the dye to leak into the cell. The wild type and the ΔCpsA strains show cocci defined as an open circle, indicating the stain only attached to the cell wall (Figure 4). However, the ΔPsr and ΔCpsA-ΔPsr mutant strains show cocci that have a stain completely filling the interior of the cell, indicating that the stain has leaked inside the cell. Additionally, we observed areas in which cocci appeared to be deformed in both ΔPsr and ΔCpsA-ΔPsr strains, suggesting that cell wall integrity defects may cause morphology changes (Figure 4). 

### 3.5. Loss of Psr Results in Increased Autolysis 

**Figure 5:**
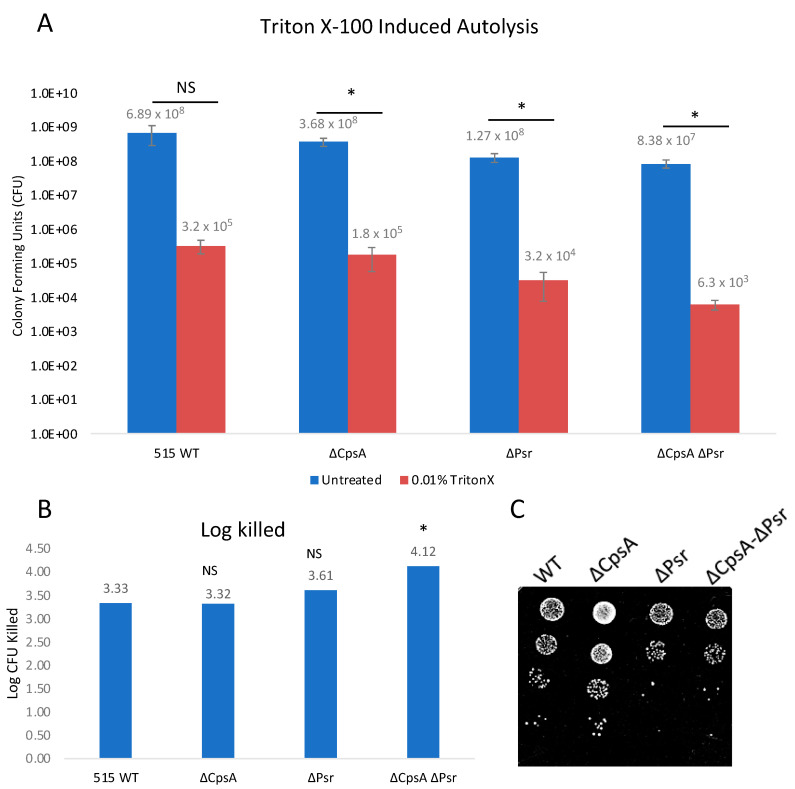
GBS autolysis increases in the presence of Triton X-100. GBS strains were grown to mid-log in THYB, normalized to OD600: 0.3 in PBS and treated with 0.01% Triton X-100 for 2 h. (**A**) Treated (Red) and untreated (Blue) samples were then serially diluted and plated on THY plates. Statistical analysis represents significance between untreated and treated cells for each strain. (**B**) Log CFU killed was calculated for each sample by taking log10 (untreated/treated). Significance was determined by comparing log CFU killed of each strain to that of wild type GBS. (**C**) 5 μL of serial dilutions of treated cells was dotted on THY plates and grown overnight for visual representation of treatment outcomes. Values represented are averages of six biological replicates. * *p* < 0.05.

Since we observed a possible change in the cell wall integrity with the fluorescent vancomycin assay, we wanted to determine if a loss of the Psr protein would make the cells more susceptible to autolysis. Triton X-100, a typical non-ionic detergent that works by breaking lipid–lipid or lipid–protein associations, was used to examine the integrity of the cell walls of the four GBS strains. Exposure of log phase growth cells to 0.01% Triton X-100 for 2 h resulted in the ΔPsr and ΔCpsA-ΔPsr mutant strains revealing an increased sensitivity to Triton-X-100-induced autolysis (Figure 5A). Log killed values were determined by comparing the final growth of the untreated culture to the final growth of the culture with treatment of the same strain, thus accounting for the differences in the growth of each strain. The ΔPsr mutant and ΔCpsA-ΔPsr mutant strains demonstrated a log killing of 3.61 and 4.12, respectively, compared to 3.33 and 3.32 for the wild type and ΔCpsA strains (Figure 5B). Therefore, the simultaneous absence of CpsA and Psr results in a significant decrease (*p* < 0.05) in cell integrity when exposed to Triton X-100.

**Figure 6:**
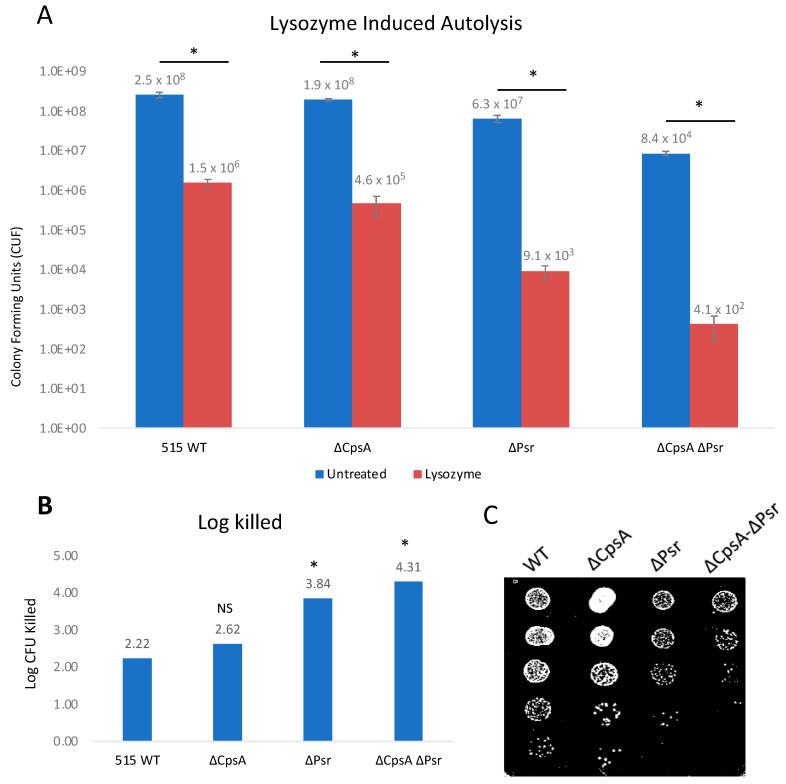
GBS autolysis increases in the presence of lysozyme. GBS strains were grown to mid-log in THYB, normalized to OD600: 0.3 in PBS and treated with 1 mg/mL lysozyme for 24 h. (**A**) Treated (Red) and untreated (Blue) samples were then serially diluted and plated on THY plates and counted. Statistical analysis represents significance between untreated and treated cells for each strain. (**B**) Log CFU killed was calculated for each sample taking Log10 (untreated/treated). Significance was determined by comparing log CFU killed of each strain to that of wild type GBS. (**C**) 5 μL of serial dilutions of treated cells was dotted on THY plates and grown overnight for visual representation of treatment outcomes. Values represented are averages of four biological replicates. * *p* < 0.05.

A second method used to examine the cell wall integrity was an exposure of all strains to lysozyme. Lysozyme cleaves peptidoglycan in bacterial cell walls by catalyzing the hydrolysis of β-(1,4) linkages between the N-acetylmuramic acid (NAM) and N-acetylglucosamine (NAG) saccharides. The treatment of log phase growth cultures with 1 mg/mL lysozyme for 24 h demonstrated that lysozyme has an effect on all four strains (Figure 6A). However, the ΔPsr and ΔCpsA-ΔPsr mutant strains are significantly (*p* < 0.05) more sensitive to lysozyme (Figure 6B). As with the Triton X-100 treatment above, log killed values were determined by comparing the treated to untreated growth of each strain against itself to account for the difference in strain growth. Lysozyme treatment revealed log killed values of 3.84 and 4.31 for the ΔPsr and ΔCpsA-ΔPsr mutant strains, respectively, compared to 2.22 and 2.62 for the wild type and ΔCpsA strains, demonstrating that the absence of the Psr protein results in a loss of cell wall integrity (Figure 6B). 

### 3.6. Absence of CpsA and Psr Result in Increased Susceptibility to Penicillin

**Figure 7:**
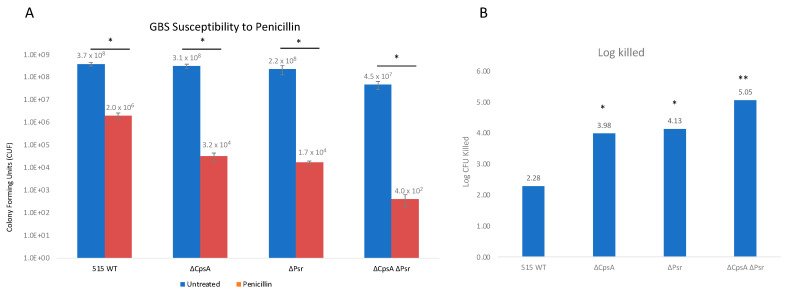
GBS susceptibility to penicillin increases in the absence of CpsA and Psr. GBS strains were grown in THY medium, normalized to OD600: 0.01 in PBS and treated with 3 μg/mL penicillin for 24 h. (**A**) Treated (Red) and untreated (Blue) samples were then serially diluted and plated on THY plates and counted. Statistical analysis represents significance between untreated and treated cells for each strain. (**B**) Log CFU killed was calculated for each sample taking Log10 (untreated/treated). Significance was determined by comparing log CFU killed of each strain to that of wild type GBS. Values represented are averages of four biological replicates. * *p* < 0.05, ** *p* < 0.005.

LCP proteins have been previously shown to have an effect on antibiotic susceptibility in Gram-positive bacteria [28]; therefore, the effect of Psr protein absence on penicillin susceptibility was examined. Penicillin is a β-lactam antibiotic and is one of the main antibiotics used to treat GBS infections. Assay results demonstrate that, in the absence of CpsA and Psr, GBS becomes more susceptible to penicillin compared to wild type GBS (Figure 7). The log killed values were 3.98 and 4.13 for ΔCpsA and ΔPsr mutants, respectively, which are significantly more (*p* < 0.05) than that of the wild type strain at 2.28. The ΔCpsA-ΔPsr double mutant demonstrates the highest amount of cell death, with a log killed value of 5.05, after penicillin exposure (*p* < 0.005).

### 3.7. Absence of CpsA and Psr Results in Decreased Capsule Levels on the Cell

**Figure 8:**
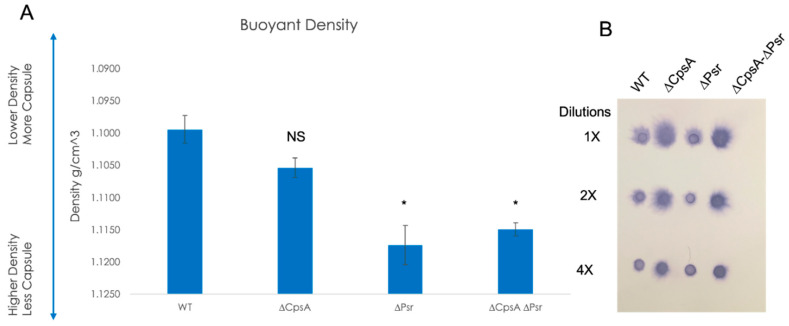
Capsule on the cell surface decreases in the absence of LCP proteins. (**A**) Percoll buoyant density centrifugation revealed less capsule in ΔCpsA, ΔPsr and ΔCpsA-ΔPsr strains in comparison to WT GBS. (**B**) Dot blot analysis on supernatants of WT, ΔCpsA, ΔPsr and ΔCpsA-ΔPsr strains reveals that more supernatant is released into the supernatant of ΔCpsA and ΔCpsA-ΔPsr strains. Significance was determined by comparing densities of each strain to that of wild type GBS. Values represented are averages of three biological replicates. * *p* < 0.05.

Previous research confirms that the LCP family protein, CpsA, plays a role in the amount of capsule on the cell, as well as binding specifically to the capsule promoter [22], and in the attachment of the capsular polysaccharide to the peptidoglycan cell wall [19,23,29]. To determine if the LCP protein, Psr, also has an effect on the amount of capsule on the cell, Percoll density gradient centrifugation was performed, as reported in previous research with GBS CpsA [22,30]. Buoyant density centrifugation sediments cells based on their buoyancy. For an examination of the capsule level, the more capsule produced, the less dense or more buoyant the cells. However, cells with less capsule will result in a higher density or less buoyancy. Results confirmed that capsule levels decreased in the absence of CpsA as shown previously, but also in the absence of Psr, and the ΔCpsA-ΔPsr double mutant (Figure 8A). Of note, a greater reduction in the capsule level was observed in the ΔPsr mutant strain than in that of the ΔCpsA mutant strain. However, the double mutant ΔCpsA-ΔPsr strain and the ΔPsr strain both showed a significant capsule reduction in comparison to the wild type strain (Figure 8A). 

To determine whether the capsule reduction is due to a reduction in the overall capsule production or due to the capsule being released into the supernatant, a dot blot analysis was performed on supernatants of the four GBS strains. The dot blot results showed the capsule being released into the supernatant in the ΔCpsA and the ΔCpsA-ΔPsr double mutant strains, but not in the case of the wild type and ΔPsr strains (Figure 8B), confirming that CpsA functions as a ligase that attaches the capsule onto the cell wall. The buoyant density does not depend on the chain length, as experiments using sonicated cultures to reduce all chains to the same length did not demonstrate a significant change in the density (supplemental Figure S1).

### 3.8. Absence of CpsA and Psr Results in a Decrease in GBS Virulence In Vivo

**Figure 9:**
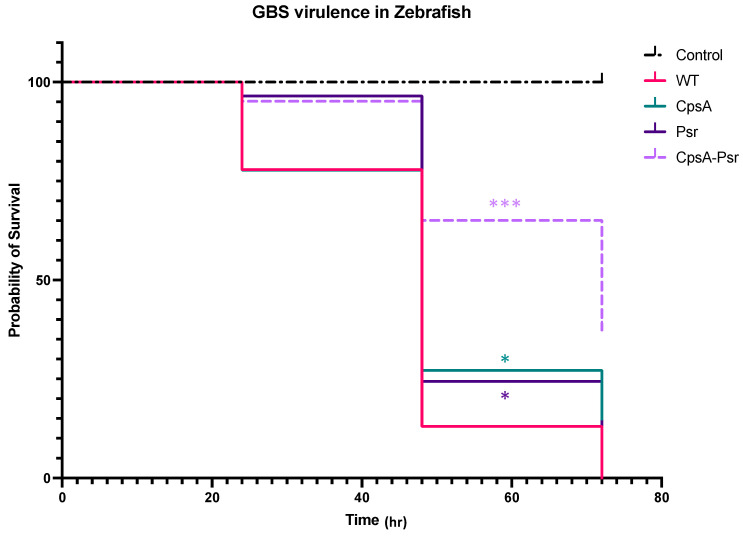
CpsA-Psr double mutant results in decreased virulence on GBS in vivo. GBS virulence was examined in vivo by injecting the yolk sacs of zebrafish 3 days post-fertilization with 100 CFU of WT, ΔCpsA, ΔPsr and ΔCpsA-ΔPsr mutant strains. Values represented are averages of four biological replicates, with a minimum of 105 total zebrafish injected per strain. * *p* < 0.05, *** *p* < 0.0005.

Previous studies have suggested that LCP proteins are important in the virulence of Gram-positive bacteria [22,30,31]. In order to determine whether the absence of the Psr protein results in increased recognition by the host immune system and a subsequently decreased virulence, the zebrafish infectious disease model was used to determine the virulence of the four GBS strains. Zebrafish larvae were injected into the yolk sac at three days post-fertilization with 100 CFU of each GBS strain, and were monitored over 72 h. The results confirmed the virulence of the wild type GBS strain, as the zebrafish injected with this strain had a 0% percent survival rate by 72 h. All three mutant GBS strains demonstrated various levels of attenuation, with a 13% survival rate in the ΔCpsA-injected group, 8% survival rate in the ΔPsr-injected group and 32% survival rate in the ΔCpsA-ΔPsr-double-mutant-injected zebrafish (Figure 9).

## 4. Discussion

The capsular polysaccharide and peptidoglycan cell wall help to protect the bacterial cell from multiple environmental factors that it may encounter within the host, as well as serving as a shield against the host immune system. LCP family proteins have been found to be crucial to the proper formation and assembly of the cell wall and capsule in several bacterial species [22,23,31,32,33]. Consistent with previous findings, GBS LCP family proteins examined in this study play an important role in the synthesis and maintenance of the cell wall and cell-wall-associated capsule. 

The slow growth rate of the ΔPsr and ΔCpsA-ΔPsr mutant strains of GBS in comparison to the wild type strain indicate that the Psr protein is an important housekeeping protein that is required for the proper growth of the GBS cell. The results from the cell viability assay (live/dead assay) indicate that the slow growth rate of ΔPsr and ΔCpsA-ΔPsr mutant strains is not due to an increased cell death in these strains during the early time frame of the growth curve (~3–5 h), as the cell viability is negligibly affected during the same time period, as shown by the live/dead assay during the log phase of growth. However, after reaching a stationary phase and growing overnight (~24 h), the ΔCpsA-ΔPsr double mutant strain exhibited a 41% cell death. This suggests that Psr is required for both normal cell growth and cell viability over time. Moreover, morphological changes, such as longer chain lengths and larger, swollen cocci observed in the cells of ΔPsr and ΔCpsA-ΔPsr strains, support the hypothesis that the Psr protein plays an important role in cell wall synthesis, and that, in the absence of Psr, the cell cannot maintain proper shape. An inconsistent cell shape was also observed in LCP-deficient strains of *S. aureus* [19] and *S. mutans* [17,32,33,34], along with effects on cell division. Transmission electron microscopy (TEM) analysis on the ΔPsr and ΔCpsA-ΔPsr mutant strains will need to be carried out to confirm cell division defects due to these mutations in GBS. 

LCP protein deficiencies have also been linked to increased autolysis in bacteria. For instance, in *S. mutans*, Psr- and BrpA-deficient strains demonstrated increased autolysis after exposure to Triton X-100 [17]. Similarly, in *S. aureus*, LCP mutants demonstrated a greater susceptibility to autolysis after being treated with Triton X-100 [31]. In this study, we examined the effect of sub-inhibitory concentrations of Triton X-100 and lysozyme on the mutant strains and observed increased autolysis in GBS when the Psr-deficient strains were exposed to these reagents. These results are supported by previous findings that linked LCP and related proteins to autolysis in *S. aureus* [31,35], *S. pneumoniae* [36] and *S. mutans* [17]. Since lysozyme affects the linkage between NAM and NAG in the peptidoglycan cell wall [37] where LCP proteins also function, our results suggest that, in the absence of Psr, other LCP family proteins, such as CpsA, can partially compensate for that function, as demonstrated by the double mutant being the most sensitive strain to lysozyme exposure. 

β-lactam antibiotics such as penicillin are the first line of treatment used to treat pregnant women during delivery that are known to be colonized with GBS [13]. Penicillin works by inhibiting the binding of transpeptidases, such as penicillin binding proteins and LCP proteins, that fuse cell wall peptidoglycan chains to one another. The penicillin susceptibility assay conducted in this study shows that, in the absence of one LCP protein, GBS becomes more susceptible to penicillin, suggesting that the CpsA and Psr proteins both play a role in protecting the cell from penicillin. The significant increase in penicillin susceptibility in the ΔCpsA-ΔPsr double mutant strain suggests that LCP proteins have the ability to partially compensate for the functions of other LCP proteins when they are missing. Similar antibiotic susceptibility assays have been performed in *S. aureus*, where the deletion of the LcpC protein resulted in increases in susceptibility to β-lactam antibiotics, including penicillin [28].

We previously demonstrated that the loss of the CpsA protein in GBS leads to decreased capsule levels [22] similar to that shown for a Cps2A deletion of *S. pneumoniae* [18]. Results from the buoyant density centrifugation experiments in this study revealed that the ΔPsr and ΔCpsA-ΔPsr mutant strains have an increased density compared to the wild type strain, suggesting that the loss of the Psr protein also leads to decreased capsule on the cell surface of GBS. Of note is the presence of excess capsule in the supernatant of the ΔCpsA mutant strain, as well as the ΔCpsA-ΔPsr double mutant strain. This is expected, as CpsA has been proposed to be the ligase that attaches the polysaccharide capsule to the cell wall [18,23]. However, what was not expected was what appears to be the absence of excess capsule in the supernatant (although not quantitative) of the ΔPsr mutant strain, even though the buoyant density assay shows less capsule on the cell surface in this mutant strain. This suggests that the Psr protein also has an effect on the capsule quantity, but it is not through the same mechanism as that of the CpsA protein. A loss of buoyancy was not due to the extra long chains of cocci, as sonicated cultures yielded similar buoyancy levels to untreated cultures that exhibited longer chain lengths and cell aggregation (supplementary data). 

The in vivo virulence assay results demonstrate that, in the absence of either the CpsA or Psr proteins, the GBS virulence decreases significantly, but the most significant attenuation is observed in zebrafish that have been injected with the ΔCpsA-ΔPsr double mutant strain. The striking difference with the increased attenuation of the double mutant strain, compared to the attenuation of the single mutant strains, suggests that there is some redundancy in the function of the proteins in vivo. The presence of one protein can partially rescue the virulence in the absence of the other, but when both proteins are absent, the virulence is greatly attenuated. Studies involving BrpA-deficient GBS also indicate decreased attenuation in a mouse model of infection, as well as in human whole blood [24]. Additionally, in vivo studies in *S. aureus* indicate that mice infected with a LcpC-deficient strain have a higher survival rate in comparison to those infected with the wild type strain [28]. In this study, the increased survival observed with the zebrafish infected with the LCP mutants may be due to several factors. Since the strains that are missing the Psr protein, especially the ΔCpsA-ΔPsr double mutant strain, do not survive well in culture over time, it can be speculated that these mutants also have decreased viability overtime in vivo, and do not survive as long at the wild type strain. Another explanation may be that the decreased cell wall integrity observed in the ΔPsr and the ΔCpsA -ΔPsr mutant strains makes them more susceptible to host defense systems used to eliminate invading pathogens. In addition, the lower capsule levels on the cell surface of the mutant strains make those cells more susceptible to phagocytosis from the host immune system.

One consistency observed throughout this study was that the ΔCpsA-ΔPsr double mutant strain showed a greater deficiency compared to the other three strains analyzed, including the overall growth rate, sensitivity to autolysis, viability over time and virulence in vivo, suggesting that, similar to other Gram-positive bacteria, LCP proteins have partial redundancy in function. Previous studies in *S. aureus* [31] and *S. mutans* [17] also show that, in the absence of one LCP protein, other family members often compensate for the missing protein; however, double mutants are either non-viable or significantly more susceptible to the assays compared to the wild type or single LCP mutant. 

Overall, the data presented here demonstrate the importance of a resilient cell envelope surrounding GBS for long-term viability and protection from environmental factors, including the host immune system. All LCP family proteins are transmembrane proteins, with the majority of the protein being in the extracellular environment, making them excellent targets for new drug therapies. Our results demonstrate the key role played by Psr in providing a robust cell envelope; therefore, it could be exploited as a possible target for new therapeutic strategies for treating GBS infections.

## Supplementary Materials

The following are available online at https://www.mdpi.com/article/10.3390/microorganisms10020217/s1, Figure S1: Creating shorter chains by sonication does not affect buoyancy.
