# Supplementary material for: The LCP Family Protein, Psr, Is Required for Cell Wall Integrity and Virulence in Streptococcus agalactiae
Source: Microorganisms. 2022 Jan 20;10(2):217. doi: 10.3390/microorganisms10020217 (PMC8875755; doi:10.3390/microorganisms10020217)
Supplement: Supplementary file 1 [file microorganisms-10-00217-s001.zip › microorganisms-1534357-SI.pdf]

Psr Manuscript supplemental data

Supplemental Materials and Methods

*Sonication*

Overnight cultures were sub-cultured and grown statically at 37°C. Cells were collected at mid-log growth phase and normalized to 0.3 $OD_{600}$ in PBS (phosphate buffered saline, pH 7.4). Cells were sonicated (Fisher Scientific Sonic Dismembrator 60) at level 2 (4 Watts) for 6 seconds on ice. Serial 10-fold dilutions were plated on THY-agar plates and compared to untreated cells.

Supplementary Figure S1

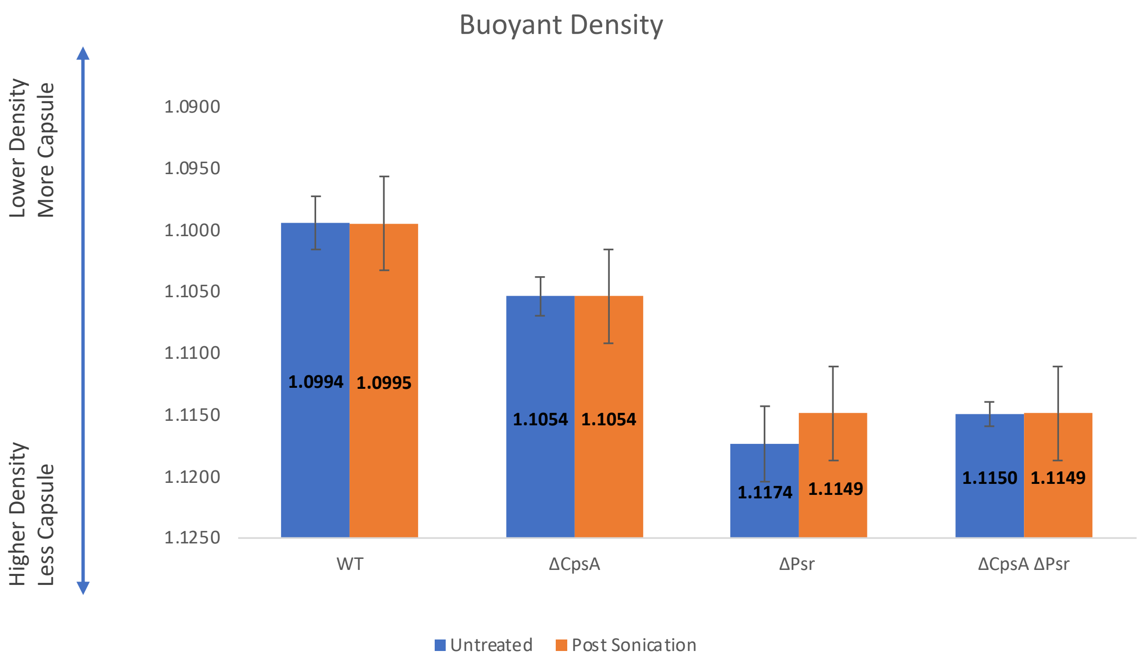


Figure S1. Creating shorter chains by sonication does not affect buoyancy**.** The cells were grown to mid-log-phase and normalized to $OD_{600}$ 0.06 were briefly sonicated in order to break the chains into smaller chains of cocci. The buoyant density assay was then performed to determine whether long chains affect the buoyancy of GBS in the mutant strains. The difference in density between sonicated and un-sonicated samples was negligible. Values represented are averages of three biological replicates.
